# Aging and Osteoarthritis: An Inevitable Encounter?

**DOI:** 10.1155/2012/950192

**Published:** 2012-06-07

**Authors:** Thomas Hügle, Jeroen Geurts, Corina Nüesch, Magdalena Müller-Gerbl, Victor Valderrabano

**Affiliations:** ^1^Osteoarthritis Research Center, Department of Orthopaedic Surgery, University Hospital Basel, Basel University, Spitalstrasse 21, 4031 Basel, Switzerland; ^2^Department of Rheumatology, University Hospital Basel, Felix-Platter-Spital, 4012 Basel, Switzerland; ^3^Institute of Anatomy, University of Basel, 4056 Basel, Switzerland

## Abstract

Osteoarthritis (OA) is a major health burden of our time. Age is the most prominent risk factor for the development and progression of OA. The mechanistic influence of aging on OA has different facets. On a molecular level, matrix proteins such as collagen or proteoglycans are modified, which alters cartilage function. Collagen cross-linking within the bone results in impaired plasticity and increased stiffness. Synovial or fat tissue, menisci but also ligaments and muscles play an important role in the pathogenesis of OA. In the elderly, sarcopenia or other causes of muscle atrophy are frequently encountered, leading to a decreased stability of the joint. Inflammation in form of cellular infiltration of synovial tissue or subchondral bone and expression of inflammatory cytokines is more and more recognized as trigger of OA. It has been demonstrated that joint movement can exhibit anti-inflammatory mechanisms. Therefore physical activity or physiotherapy in the elderly should be encouraged, also in order to increase the muscle mass. A reduced stem cell capacity in the elderly is likely associated with a decrease of repair mechanisms of the musculoskeletal system. New treatment strategies, for example with mesenchymal stem cells (MSC) are investigated, despite clear evidence for their efficacy is lacking.

## 1. Introduction

**Figure 1:**
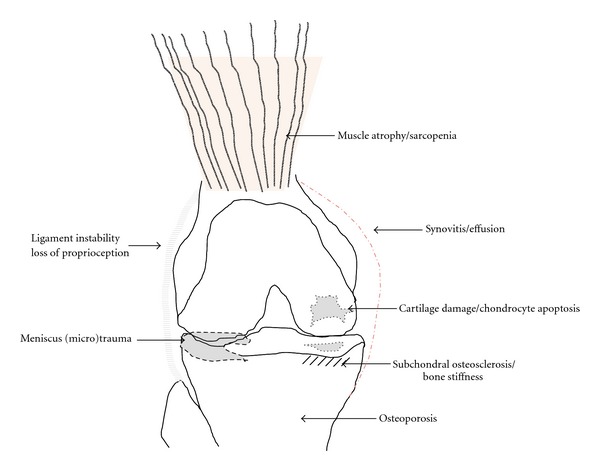
Osteoarthritis as a whole joint disease in the elderly.

Half of all persons aged over 65 suffer from osteoarthritis (OA) [1]. As a matter of fact, age is the most prominent risk factor for the initiation and progression of OA. The common explanation for this is the cumulative effect of mechanical load over the years, resulting clinically in “wear and tear” and pathologically in cartilage breakdown [2]. Therefore, OA has been regarded as a naturally occurring, irreversible disorder, rather than a specific, potentially treatable disease. During the last decade, however, it became clearer that OA is not a purely mechanical problem. Inflammatory and metabolic processes are substantially involved in the pathogenesis and progression of OA. Not only cartilage, but also subchondral bone, menisci, muscles as well as fat, and synovial tissues play an important role, notably in the early phase of OA (Figure 1). Therefore, OA has been referred to as a “whole joint disease.” Despite a higher complexity, this concept has not only improved our understanding of the disease but also indicates potentially new treatment strategies.

To understand why aging predisposes to the development of OA, a link between aging processes and the pathological changes in the OA joint needs to be established. On a molecular level, aging research has revealed intrinsic changes in the structure of extracellular matrix proteins such as collagen or proteoglycans. Stiffening of the collagen network or increased glycation provoke a functional impairment of cartilage and joint function [3]. Aging also has profound effects on cellular processes notably leading to enhanced apoptosis and reduced cellular regeneration [4].

Nonenzymatic collagen cross-linking leads abnormalities in bone toughness and stiffness. Bone plasticity is further suppressed by an increase of osteon density, which leads to a lower potency of crack-bridging mechanisms [5].

Synovitis is frequently involved in OA, notably in the early phase of the disease [6]. This has been demonstrated both by histological studies and magnetic resonance imaging (MRI) analyses [7]. OA synovial fluid contains proinflammatory cytokines. This is demonstrated clinically when a ruptured baker cyst leads to painful swelling of the surrounding soft tissue. Similarly, the penetration of synovial fluid into the subchondral space after microfracture of the subchondral bone plate can induce an inflammatory response.

The exact reason for the inflamed status of the OA joint remains unclear. Overuse can lead to an activation of osteoblasts but also mast cells. Subsequently, other immune cells of the innate or adaptive immune system are attracted. Crystals, for example, in calcium pyrophosphate disease, a typical bystander in OA can activate the inflammasome leading to interleukin-1 activation [8]. On the other hand, during aging the immune system is less capable to resolve inflammation in general. In other words, not only the initiation of inflammation, but also the lack of inflammation might be involved in OA, notably in the aged individual.

Notwithstanding, mechanical dysfunction is still regarded as key player in OA pathogenesis, predominantly in weight bearing joints. This is also referred as “continuous loading theory.” Apart from the aforementioned molecular impairment of cartilage, also the musculature is involved in stabilization and therefore protection of the joint. Muscle atrophy is well recognized in OA pathogenesis [9]. In the elderly, muscle atrophy is highly prevalent; this can be caused by sarcopenia in general, lack of physical training, malnutrition or arthrogenic muscle inhibition as a direct consequence of OA.

Finally, aging leads to a reduced regenerative capacity, for example, shown by the reduced levels of stem cells in connective tissue in the elderly. The elderly enters a catabolic state, where a presenescent cell state leads to subsequent loss of connective tissue homeostasis.

In this paper we give an overview of age-related structural and functional changes occurring in the course of OA.

## 2. Changes in Gait Biomechanics in OA

Changes in the joint biomechanics of patients suffering from OA have been shown in various studies. Gait analyses showed that patients with OA in the medial compartment of the knee walk with a more extended knee at heel strike [10]. Additionally, the external knee adduction moment has been identified as an important factor in medial knee OA [10–12]. The peak knee adduction moment has been shown to increase with increasing varus malalignment [12] and with increasing radiographic disease severity [10, 11]. Although the knee adduction moment has been identified as a risk factor for disease progression [11], there is also evidence that in early stages of OA, patients are able to reduce the knee adduction moment by moving the trunk laterally [10]. However, to achieve this, sufficient hip abduction muscle strength is needed to balance the external hip adduction moment.

In a recent meta-analysis, Pietrosimone et al. [13] showed that patients with knee OA have a deficit in the activation of the quadriceps muscles. Additionally, there is evidence that weak knee extensor increases the risk to develop symptomatic knee OA [14, 15]. The muscles surrounding the knee joint are important for the stability. Muscle weakness can therefore impair the neuromuscular protection of the joint leading to microtraumas and possibly joint damage [16]. Hence, muscular dysfunction might not only be a consequence of the disease but could also play a role in the development of OA [14, 16].

## 3. Biomechanical Changes in the Elderly

Walking speed is relatively consistent between age 20 and 70 and declines after age 70 [17, 18]. Older people often walk with a reduced step length which is a possible adaptation to lower muscle strength and fear of tripping and falling [19, 20]. In this study it has been shown that patients with knee OA produce lower knee extension forces than age-matched controls. These force levels were comparable to those of an on average 20 years older age group. In contrast to the knee OA patients that also showed joint laxity, the healthy elderly could maintain normal joint biomechanics during walking by increasing the muscle activity. Additionally, aging affects the joint proprioception. In older subjects the joint position sense was worse than in young ones. It was also seen that knee OA patients have an even worse joint position sense than healthy elderly of a similar age. This could indicate that the age-related decline in proprioception increases the risk of OA due to reduced joint stability and increased joint stress [21].

While some age-related changes in the gait pattern like a reduced walking speed could be beneficial in reducing the risk or progression of OA [10], other changes, like muscle weakness or reduced proprioception, could increase the risk of OA, especially in the presence of additional factors such as joint laxity or pain.

Obesity has a negative impact on biomechanics, at least in weight bearing joints [22] and has been demonstrated as a risk factor for OA in large studies [23]. Interestingly, the negative effect of obesity on cartilage decay is reversible. A recent study postulated an improved cartilage quality in the medial compartment in patients with knee OA after weight loss [24]. In the elderly, obesity in combination with sarcopenia is frequently encountered, a combination which has further negative influence on OA [25]. Whether obesity causes sarcopenia in the elderly, or obesity is a consequence of reduced physical activity, remains a chicken-egg question.

## 4. Cartilage Decay with Age

Progressive loss of articular cartilage is considered as the hallmark of OA. Cartilage consists of chondrocytes that are embedded in a specialized extracellular matrix (ECM), which is composed of water, collagen II, and proteoglycans. Chondrocytes are quiescent nondividing cells responsible for the maintenance of cartilage homeostasis through a balanced production of catabolic and anabolic factors. During aging, senescence is induced either by progressive telomere shortening due to repeated cell division or environmental stress factors, such as oxidative damage, chronic inflammation or ultraviolet radiation [26]. Indeed, in vitro proliferation of chondrocytes from young donors differs dramatically from aged and OA donors, due to a complete lack of cell division in the latter [27]. However, as chondrocyte proliferation is scarcely observed in normal or osteoarthritic cartilage, telomere shortening is not regarded as the most likely mechanism for senescence [28]. Accumulation of oxidative damage is considered as a major player in the induction of senescence in chondrocytes. Decreased expression of oxygen radical scavengers, such as superoxide dismutase, has been demonstrated in OA cartilage [29]. There is ample evidence that age-dependent alterations in chondrocyte metabolism or signal transduction associates with progression of OA [30, 31].

These epigenetic-induced changes in chondrocytes skew the balance that dictates cartilage homeostasis and induces alterations of the ECM and cartilage integrity that promote development of OA. Loss of biomechanical function of cartilage during aging has been associated with a decrease in glycosaminoglycan content [32], which is normally deposited by chondrocytes. Besides direct modulation of the ECM by chondrocytes, age-related changes in ECM components has been proposed to mediate cartilage decay. Age-dependent accumulation and consequent cross-linking of advanced glycation end products (AGE) in collagen has been shown to increase cartilage stiffness, which makes it brittle and impairs its load-absorbing function [3]. Moreover, chondrocytes express the receptor for AGE (RAGE) and engagement of the receptor induces the production of cartilage-degrading enzymes [33]. Together, age-related changes in chondrocytes and components of ECM crucially impair the function of cartilage and its capacity to cope with mechanical or environmental stress factors, which is likely to predispose to OA development (Table 1).

## 5. Subchondral Bone Adaption in Aging and the Role of Bone Mass in OA

**Figure 2:**
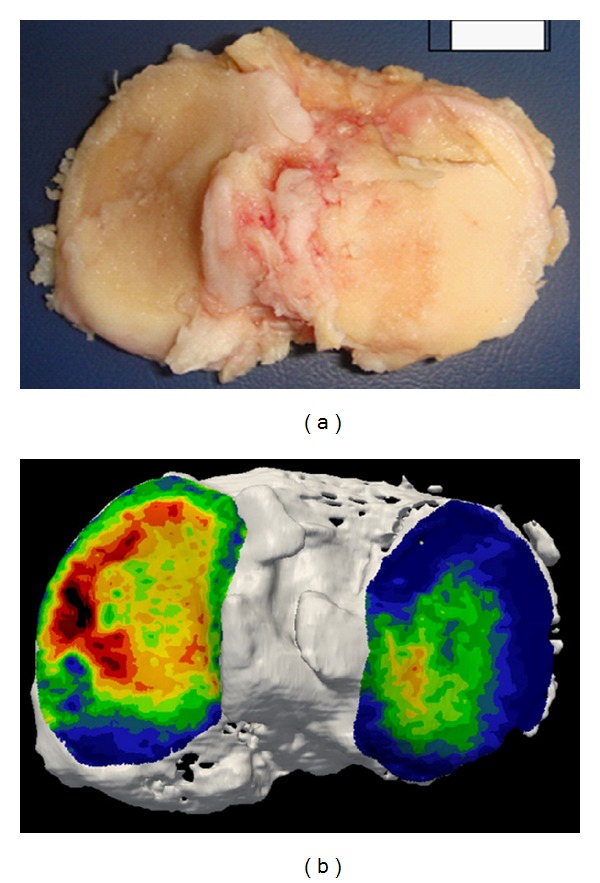
Tibial plateau of an elderly patient showing end-stage OA with complete cartilage loss (a). Computed tomography osteoabsorptiometry (CT-OAM) indicates high bone density in red (b).

The microarchitecture of the skeleton, notably the subchondral bone, constantly adapts to mechanical loading. Mechanotransduction processes lead to bone modeling and remodeling. Impaired joint loading patterns trigger high subchondral bone density, probably in order to avoid (micro) fractures and to ensure minimal joint function. Subchondral density can be accurately assessed by computed tomography osteoabsorptiometry (CT-OAM) [34].  Figure 2 shows a tibial plateau of a geriatric patient suffering from end stage knee OA who underwent joint replacement.

Complete cartilage loss is seen on the medial side. Below, CT-OAM was performed. Highest subchondral bone mineral density (BMD) indicated in red is observed in the region of maximal cartilage damage. On a molecular level*‚* mechanosensors on osteoblasts have been discovered which induce interleukin-6 and -8 expression within subchondral bone affected by OA upon cyclic mechanical stimulation [35]. This is in line with the observation that increased BMD measurement of the knee correlates with clinical OA. However, the relation between generalized bone loss or osteoporosis and OA is still controversial. The MOST study has shown that high femoral neck and whole-body BMD are associated with an increased risk of OA [36]. However, osteophytes alter BMD assessment, so it remains unclear whether increased BMD is a consequence, rather than a cause of OA. Clearly, the higher bone density in patients with OA is not associated with a lower risk of vertebral or nonvertebral fractures [37].

## 6. Muscle Atrophy and Its Role on OA in the Elderly

Muscle atrophy can have several causes. Immobilization, malnutrition, myopathy, neurologic disorders, comorbidity of several common diseases such as cancer, congestive heart failure, COPD, or any state of chronic inflammation affect the muscle mass substantially. Also endocrinologic disorders which are generally more frequently observed in the elderly can trigger muscle atrophy, for example, low testosteron levels, diabetes mellitus, or hypothyroidism.

In contrast to secondary muscle atrophy, the commonly observed loss of muscle mass in the elderly is a syndrome referred to as “sarcopenia” [38]. Reduced muscle tissue usually starts at the age of 50 years and results in diminished muscle function. Subsequently, decreased muscle strength of the joints contribute to the pathogenesis of OA within the next decades. Fifty percent of all individuals aged over 75 suffer from sarcopenia.

The mechanistic association between OA and muscle atrophy has been clearly demonstrated. Several factors such as reduced stability, joint immobilization, restriction of range of motion but also so called arthrogenous muscle inhibition or reflex atrophy are implicated in this process. The latter is due to abnormal nociceptive afferent feedback processes, releasing neuromodulators in the spinal cord, which in turn cause a change in alpha-motoneuron excitability. In knee OA, it has been shown that quadriceps muscle strength is reduced between 20 and 40% for isometric and isokinetic contractions [39].

## 7. Physical Activity Preventing OA

Physiotherapy is the gold standard for OA treatment. The positive effect of physiotherapy on knee OA has been clearly demonstrated by a meta-analysis of 32 controlled trials [40]. Furthermore, physical activity seems to have preventive effects on OA. A consensus stating that performing structured strengthening exercises has a favorable effect on pain and functioning in sedentary patients with knee OA has been formulated by several organisations, for example, by the MOVE consensus [41]. In first line, physical activity in OA patients encounters clinical or subclinical muscle atrophy which has implications on joint stability [42], but also stimulates weight reduction [43].

## 8. The Role of Immunosenescence in OA

In the last decade, inflammation in OA has attracted increasing interest. A growing arsenal of anti-inflammatory compounds now is available which successfully works in inflammatory arthritis. On the search for disease modifying osteoarthritis drugs (DMOADs), some of those compounds have also been successfully applied to OA, at least in case reports or case series [44]. Inflammation in OA is often encountered clinically by swelling of the joint, redness and night pain. This “disease activation” in OA typically occurs in episodes, either resolving spontaneously, or sometimes by the intra-articular injection of steroids [45]. The efficacy of steroids in activated OA is well documented and safe, if applied under sterile conditions and in right intervals.

Synovial infiltration by inflammatory cells such as macrophages, mast cells or lymphocytes occurs in around 50% of the patients [6]. In MRI studies, synovitis shown with or without contrast agent correlates with pain and also radiological progression of OA. The inflammatory status of the joint is also reflected by increased pro-inflammatory cytokine levels such as TNF-alpha or IL-1 [46]. This is likely a result of the inflammatory cell infiltration in the joint and mast cell activation. Interestingly, recent findings indicate that mast cells, but also subchondral osteoblasts express mechanoreceptors which change their expression pattern upon mechanical stimulation, for example, by the expression of IL-8 [35]. But also synovial fibroblasts and even chondrocytes produce more proinflammatory cytokines and participate in inflammation.

**Figure 3:**
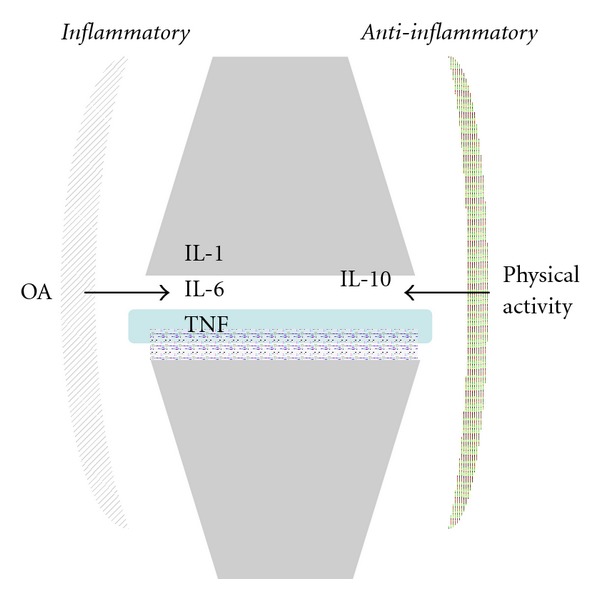
Cytokine expression at rest and during physical activity. OA: osteoarthritis, IL: interleukin, TNF: tumor necrosis factor.

In animal models, it has been shown that exercise leads to an increased expression of interleukin-10 in the joint [47]. IL-10 is a strong anti-inflammatory mediator and therefore likely implicated in the resolution of mechanically induced inflammation or irritation (Figure 3).

Immunosenescence is characterized by a reduced capacity of immune cells to encounter antigens and resolution of inflammation.

Recent data showed that physical activity indeed counteracts immunoscenesence [48]. Although the exact mechanism is still unclear, exercise impacts multiple aspects of the immune response including T-cell phenotype, T-cell proliferation, and as for IL-10, cytokine expression [49].

## 9. Cell Renewal and Stem Cell Capacity in OA

Cartilage has a low regeneration capacity due to lack of viable progenitor cells. Mesenchymal stem cells are postulated to be involved in connective tissue homeostasis and repair. The failure of repair mechanisms in connective tissues in OA is compelling. Indeed, reduced numbers of multipotent mesenchymal progenitor CD105+/CD166+ cells have been detected in the elderly. Furthermore, a lower number and lower chondrogenic and osteogenic differentiation capacity in progenitors cells from aged individuals have been demonstrated [50, 51]. Multipotent stem cells were also isolated from human cruciate ligaments suggesting their regenerative capacity also occurring in ligament structures [52]. Increased numbers of MCS are seen in the synovial fluid of patients with OA indicating a biological response [53]. These observations open the way for new therapeutic options notably in the elderly by applying those cells in the joints. Current studies investigate the possibility to obtain autologous MSC from synovial tissue in OA patients [54]. On the other hand, MSC can be obtained from other tissue, for example, adipose tissue. Notwithstanding, controlled clinical trials showing a clear efficacy of MSC treatment in OA, are still lacking.

## 10. Conclusion

Aging alters the human musculoskeletal system on a molecular and functional levels. Cellular renewal, matrix modification, and immunosenescence affect the regeneration capacity of connective tissue in general, but notably of bone and cartilage. This explains the important role of aging in the development and progression of OA. On the other side, several features observed during aging such as muscle atrophy, BMD, and inflammation are potentially reversible and should be counteracted, regardless of age.

## Figures and Tables

**Table 1:** Age-related mechanisms triggering OA.

Mechanism	Consequence
Oxidative stress	Cell senescence
Apoptosis/autophagy	Reduced regeneration capacity
Matrix protein modification (e.g., glycosylation)	Reduced elasticity, fluid content, stability
Nonenzymatic collagen cross-linking	Impaired crack-bridging potency
Sarcopenia	Reduced joint stability
Loss of proprioception	Microtrauma, Charcot-like arthropathy
Joint laxity	Microtrauma
Synovitis	Inflammatory cytokine production
Increased osteon density	Bone stiffness
Reduced circulating progenitor cells (e.g., MSC)	Impaired regeneration capacity

## References

[1] Bijlsma JW, Berenbaum F, Lafeber FP (2011). Osteoarthritis: an update with relevance for clinical practice. *The Lancet*.

[2] Aigner T, Rose J, Martin J, Buckwalter J (2004). Aging theories of primary osteoarthritis: from epidemiology to molecular biology. *Rejuvenation Research*.

[3] Verzijl N, DeGroot J, Ben ZC (2002). Crosslinking by advanced glycation end products increases the stiffness of the collagen network in human articular cartilage: a possible mechanism through which age is a risk factor for osteoarthritis. *Arthritis & Rheumatism*.

[4] Martin JA, Ellerbroek SM, Buckwalter JA (1997). Age-related decline in chondrocyte response to insulin-like growth factor-I: the role of growth factor binding proteins. *Journal of Orthopaedic Research*.

[5] Zimmermann EA, Schaible E, Bale H (2011). Age-related changes in the plasticity and toughness of human cortical bone at multiple length scales. *Proceedings of the National Academy of Sciences of the United States of America*.

[6] Sellam J, Berenbaum F (2010). The role of synovitis in pathophysiology and clinical symptoms of osteoarthritis. *Nature Reviews Rheumatology*.

[7] Benito MJ, Veale DJ, FitzGerald O, van den Berg WB, Bresnihan B (2005). Synovial tissue inflammation in early and late osteoarthritis. *Annals of the Rheumatic Diseases*.

[8] Jin C, Frayssinet P, Pelker R (2011). NLRP3 inflammasome plays a critical role in the pathogenesis of hydroxyapatite-associated arthropathy. *Proceedings of the National Academy of Sciences of the United States of America*.

[9] Valderrabano V, von Tscharner V, Nigg BM (2006). Lower leg muscle atrophy in ankle osteoarthritis. *Journal of Orthopaedic Research*.

[10] Mundermann A, Dyrby CO, Andriacchi TP (2005). Secondary gait changes in patients with medial compartment knee osteoarthritis: increased load at the ankle, knee, and hip during walking. *Arthritis & Rheumatism*.

[11] Miyazaki T, Wada M, Kawahara H, Sato M, Baba H, Shimada S (2002). Dynamic load at baseline can predict radiographic disease progression in medial compartment knee osteoarthritis. *Annals of the Rheumatic Diseases*.

[12] Foroughi N, Smith R, Vanwanseele B (2009). The association of external knee adduction moment with biomechanical variables in osteoarthritis: a systematic review. *Knee*.

[13] Pietrosimone BG, Hertel J, Ingersoll CD, Hart JM, Saliba SA (2011). Voluntary quadriceps activation deficits in patients with tibiofemoral osteoarthritis: a meta-analysis. *PM & R*.

[14] Roos EM, Herzog W, Block JA, Bennell KL (2011). Muscle weakness, afferent sensory dysfunction and exercise in knee osteoarthritis. *Nature Reviews Rheumatology*.

[15] Segal NA, Torner JC, Felson D (2009). Effect of thigh strength on incident radiographic and symptomatic knee osteoarthritis in a longitudinal cohort. *Arthritis Care and Research*.

[16] Hurley MV (1999). The role of muscle weakness in the pathogenesis of osteoarthritis. *Rheumatic Disease Clinics of North America*.

[17] Bohannon RW, Williams AA (2011). Normal walking speed: a descriptive meta-analysis. *Physiotherapy*.

[18] Judge JO, Davis RB, Ounpuu S (1996). Step length reductions in advanced age: the role of ankle and hip kinetics. *Journals of Gerontology A*.

[19] Menz HB, Lord SR, Fitzpatrick RC (2003). Age-related differences in walking stability. *Age and Ageing*.

[20] Rudolph KS, Schmitt LC, Lewek MD (2007). Age-related changes in strength, joint laxity, and walking patterns: are they related to knee osteoarthritis?. *Physical Therapy*.

[21] Pai YC, Rymer WZ, Chang RW, Sharma L (1997). Effect of age and osteoarthritis on knee proprioception. *Arthritis & Rheumatism*.

[22] Runhaar J, Koes BW, Clockaerts S, Bierma-Zeinstra SM (2011). A systematic review on changed biomechanics of lower extremities in obese individuals: a possible role in development of osteoarthritis. *Obesity Reviews*.

[23] Felson DT, Anderson JJ, Naimark A, Walker AM, Meenan RF (1988). Obesity and knee osteoarthritis. The Framingham study. *Annals of Internal Medicine*.

[24] Anandacoomarasamy A, Leibman S, Smith G (2012). Weight loss in obese people has structure-modifying effects on medial but not on lateral knee articular cartilage. *Annals of the Rheumatic Diseases*.

[25] Waters DL, Baumgartner RN (2011). Sarcopenia and obesity. *Clinics in Geriatric Medicine*.

[26] Campisi J, D’Adda di Fagagna F (2007). Cellular senescence: when bad things happen to good cells. *Nature Reviews Molecular Cell Biology*.

[27] Dozin B, Malpeli M, Camardella L, Cancedda R, Pietrangelo A (2002). Response of young, aged and osteoarthritic human articular chondrocytes to inflammatory cytokines: molecular and cellular aspects. *Matrix Biology*.

[28] Aigner T, Hemmel M, Neureiter D (2001). Apoptotic cell death is not a widespread phenomenon in normal aging and osteoarthritis human articular knee cartilage: a study of proliferation, programmed cell death (apoptosis), and viability of chondrocytes in normal and osteoarthritic human knee cartilage. *Arthritis & Rheumatism*.

[29] Regan E, Flannelly J, Bowler R (2005). Extracellular superoxide dismutase and oxidant damage in osteoarthritis. *Arthritis & Rheumatism*.

[30] Davidson ENB, Remst DF, Vitters EL (2009). Increase in ALK1/ALK5 ratio as a cause for elevated MMP-13 expression in osteoarthritis in humans and mice. *Journal of Immunology*.

[31] Cravero JD, Carlson CS, Im HJ, Yammani RR, Long D, Loeser RF (2009). Increased expression of the Akt/PKB inhibitor TRB3 in osteoarthritic chondrocytes inhibits insulin-like growth factor 1-mediated cell survival and proteoglycan synthesis. *Arthritis & Rheumatism*.

[32] Temple MM, Bae WC, Chen MQ (2007). Age- and site-associated biomechanical weakening of human articular cartilage of the femoral condyle. *Osteoarthritis and Cartilage*.

[33] Nah SS, Choi IY, Yoo B, Kim YG, Moon HB, Lee CK (2007). Advanced glycation end products increases matrix metalloproteinase-1, -3, and -13, and TNF-*α* in human osteoarthritic chondrocytes. *The FEBS Letters*.

[34] Muller-Gerbl M, Putz R, Hodapp N, Schulte E, Wimmer B (1989). Computed tomography-osteoabsorptiometry for assessing the density distribution of subchondral bone as a measure of long-term mechanical adaptation in individual joints. *Skeletal Radiology*.

[35] Sanchez C, Deberg MA, Bellahcene A (2008). Phenotypic characterization of osteoblasts from the sclerotic zones of osteoarthritic subchondral bone. *Arthritis & Rheumatism*.

[36] Nevitt MC, Zhang Y, Javaid MK (2010). High systemic bone mineral density increases the risk of incident knee OA and joint space narrowing, but not radiographic progression of existing knee OA: the MOST study. *Annals of the Rheumatic Diseases*.

[37] Arden NK, Crozier S, Smith H (2006). Knee pain, knee osteoarthritis, and the risk of fracture. *Arthritis & Rheumatism*.

[38] Rosenberg IH (2011). Sarcopenia: origins and clinical relevance. *Clinics in Geriatric Medicine*.

[39] Hassan BS, Doherty SA, Mockett S, Doherty M (2002). Effect of pain reduction on postural sway, proprioception, and quadriceps strength in subjects with knee osteoarthritis. *Annals of the Rheumatic Diseases*.

[40] Fransen M, McConnell S (2008). Exercise for osteoarthritis of the knee. *Cochrane Database of Systematic Reviews*.

[41] Roddy E, Zhang W, Doherty M (2005). Evidence-based recommendations for the role of exercise in the management of osteoarthritis of the hip or knee—the MOVE concensus. *Rheumatology*.

[42] Slemenda C, Heilman DK, Brandt KD (1998). Reduced quadriceps strength relative to body weight: a risk factor for knee osteoarthritis in women?. *Arthritis & Rheumatism*.

[43] Davis MA, Neuhaus JM, Ettinger WH, Mueller WH (1990). Body fat distribution and osteoarthritis. *American Journal of Epidemiology*.

[44] Abramson SB, Yazici Y (2006). Biologics in development for rheumatoid arthritis: relevance to osteoarthritis. *Advanced Drug Delivery Reviews*.

[45] Hepper CT, Halvorson JJ, Duncan ST, Gregory AJ, Dunn WR, Spindler KP (2009). The efficacy and duration of intra-articular corticosteroid injection for knee osteoarthritis: a systematic review of level I studies. *Journal of the American Academy of Orthopaedic Surgeons*.

[46] Kapoor M, Martel-Pelletier J, Lajeunesse D, Pelletier JP, Fahmi H (2011). Role of proinflammatory cytokines in the pathophysiology of osteoarthritis. *Nature Reviews Rheumatology*.

[47] Helmark IC, Mikkelsen UR, Borglum J (2010). Exercise increases interleukin-10 levels both intraarticularly and peri-synovially in patients with knee osteoarthritis: a randomized controlled trial. *Arthritis Research & Therapy*.

[48] Senchina DS, Kohut ML (2007). Immunological outcomes of exercise in older adults. *Clinical Interventions in Aging*.

[49] Nieman DC, Henson DA, Gusewitch G (1993). Physical activity and immune function in elderly women. *Medicine and Science in Sports and Exercise*.

[50] Chang HX, Yang L, Li Z, Chen G, Dai G (2011). Age-related biological characterization of mesenchymal progenitor cells in human articular cartilage. *Orthopedics*.

[51] de Bari C, Kurth TB, Augello A (2010). Mesenchymal stem cells from development to postnatal joint homeostasis, aging, and disease. *Birth Defects Research C*.

[52] Cheng MT, Yang HW, Chen TH, Lee OK (2009). Isolation and characterization of multipotent stem cells from human cruciate ligaments. *Cell Proliferation*.

[53] Sekiya I, Ojima M, Suzuki S (2012). Human mesenchymal stem cells in synovial fluid increase in the knee with degenerated cartilage and osteoarthritis. *Journal of Orthopaedic Research*.

[54] Kim MJ, Son MJ, Son MY (2011). Generation of human induced pluripotent stem cells from osteoarthritis patient-derived synovial cells. *Arthritis & Rheumatism*.

